# Promotion and Upregulation of a Plasma Membrane Proton-ATPase Strategy: Principles and Applications

**DOI:** 10.3389/fpls.2021.749337

**Published:** 2021-12-22

**Authors:** Zirong Ren, Bazhen Suolang, Tadashi Fujiwara, Dan Yang, Yusuke Saijo, Toshinori Kinoshita, Yin Wang

**Affiliations:** ^1^Institute of Ecology, College of Urban and Environmental Sciences and Key Laboratory for Earth Surface Processes of Ministry of Education, Peking University, Beijing, China; ^2^Division of Biological Sciences, Nara Institute of Science and Technology, Nara, Japan; ^3^College of Urban and Environmental Sciences, Peking University, Beijing, China; ^4^Institute of Transformative Bio-Molecules (WPI-ITbM), Nagoya University, Nagoya, Japan

**Keywords:** *Arabidopsis*, environmental plasticity, plasma membrane proton ATPase, rice, stomata

## Abstract

Plasma membrane proton-ATPase (PM H^+^-ATPase) is a primary H^+^ transporter that consumes ATP *in vivo* and is a limiting factor in the blue light-induced stomatal opening signaling pathway. It was recently reported that manipulation of PM H^+^-ATPase in stomatal guard cells and other tissues greatly improved leaf photosynthesis and plant growth. In this report, we review and discuss the function of PM H^+^-ATPase in the context of the promotion and upregulation H^+^-ATPase strategy, including associated principles pertaining to enhanced stomatal opening, environmental plasticity, and potential applications in crops and nanotechnology. We highlight the great potential of the promotion and upregulation H^+^-ATPase strategy, and explain why it may be applied in many crops in the future.

## Introduction

The growth of terrestrial plants by fixing CO_2_ from the atmosphere plays an important role as a carbon sink in global change. As the world population increases year by year and urbanization intensifies, areas available for vegetation are becoming more limited. There is a pressing need to improve plant growth efficiency in order to neutralize CO_2_ emissions caused by human activities worldwide. Recent studies on the manipulation of plasma membrane proton-ATPase (PM H^+^-ATPase) in an *Arabidopsis thaliana* plant model have suggested a new strategy to enhance plant growth (Young et al., 1998; Robertson et al., 2004; Wang et al., 2014a; Hoffmann et al., 2019; Zhang et al., 2021). Because PM H^+^-ATPase is a key enzyme for regulating membrane potential and is conserved in most plant species, the promotion and upregulation of a PM-ATPase strategy are expected to be applied outside the laboratory. The current review discusses the main details of the promotion and upregulation H^+^-ATPase strategy, including the fundamentals of PM H^+^-ATPase involved in stomatal opening; evidence on how overexpression of PM H^+^-ATPase enhances plant growth, aspects of environmental plasticity associated with promotion and upregulation H^+^-ATPase, and provides examples of applications in crops and nanotechnology.

## Function of PM H^+^-ATpase

### PM H^+^-ATPase Family

**Figure 1:**
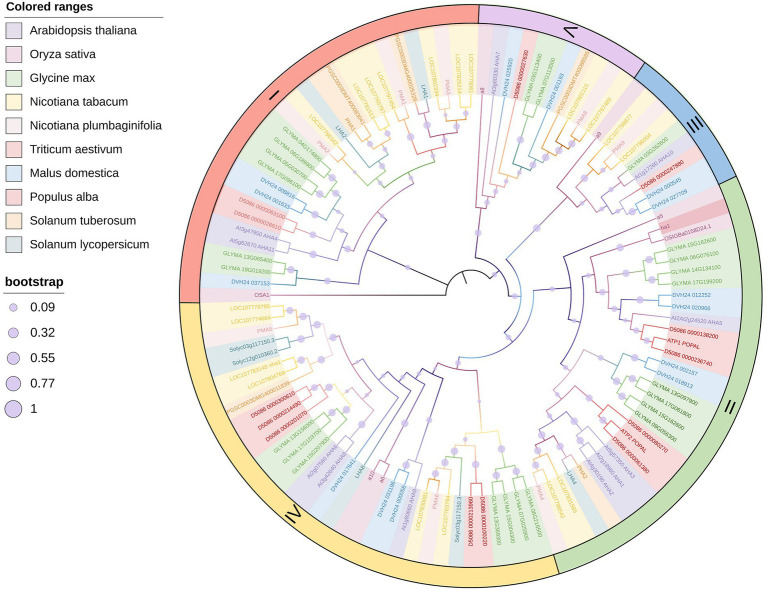
Phylogenetic tree of main crop PM H^+^-ATPases. The phylogenetic tree was constructed using PM H^+^-ATPase amino acid sequences. Different colored ranges represent different species as shown in the top legend. Different sized purple circles represent the bootstrap of every branch as shown in the bottom legend. Roman numerals indicate subfamilies defined by Arango et al. (2003). The main part of the figure was drawn by iTOL (https://itol.embl.de/).

PM H^+^-ATPase is a member of the P-type ATPase super family and is the primary active transport system in plants and fungi (Sussman, 1994). The activated state of PM H^+^-ATPase results in an electrochemical gradient that is the driving force behind the transport of other solutes across the cell membrane (Palmgren, 2001; Pedersen et al., 2018). This process involves the hyperpolarization of the plasma membrane and the hydrolysis of ATP, and these two steps occur almost simultaneously (Morsomme and Boutry, 2000). PM H^+^-ATPase extrudes H^+^ from the cell, generating a proton motive force with a membrane potential of −120 to −160 mV (negative inside) and a pH gradient of 1.5 to 2.0 units (acidic outside; Sze et al., 1999). Thus, PM H^+^-ATPase facilitates efficient transport and normal physiological functioning of the plant cell. In several species, PM H^+^-ATPase is encoded by a gene family composed of multiple genes, such as the PM H^+^-ATPase gene family of *Nicotiana tabacum* which contains nine genes (Duby and Boutry, 2009). *A. thaliana* H^+^-ATPase (AHA) is encoded by a family of 11 genes, and *Oryza sativa* H^+^-ATPase (OSA) is encoded by a family of 10 genes (Arango et al., 2003; Baxter et al., 2003; Toda et al., 2016). Distinct subtypes of the same plant may have different expression levels, expression sites, and modes of phosphorylation regulation, but there is also overlapping expression and functional redundancy. Phylogenetic analysis of the H^+^-ATPases in several primary C3 crops indicates that the evolutionary processes of different H^+^-ATPase isoforms in the same species are inconsistent (Figure 1). Notably however, all plant isoforms are included in the five subfamilies classified by Arango et al. (2003). Interestingly, the isoforms of *O. sativa* and *Triticum aestivum* were adjacent to each other but separate from other plants, suggesting a conserved H^+^-ATPase evolution between them (Figure 1).

### Structure and Regulation of PM H^+^-ATPase

Given the important role of H^+^-ATPase in plant physiology and biochemistry, many researchers have studied the molecular structure, function, and regulatory mechanisms associated with the enzyme. PM H^+^-ATPase has a molecular mass of approximately 100 kDa and is structurally highly conserved in plants and fungi (Sussman, 1994). The structures all share 10 transmembrane segments, as well as C-terminal and N-terminal domains extending into the cytosolic side of the plasma membrane (Morsomme and Boutry, 2000; Pedersen et al., 2007; Falhof et al., 2016). Autoinhibitory C-terminal domains have been identified in both plant and fungal PM H^+^-ATPases (Pedersen et al., 2018). The autoinhibitory domain is a key region with respect to the regulation of H^+^-ATPase activity (Palmgren et al., 1990; Axelsen et al., 1999; Ekberg et al., 2010). N-terminal and C-terminal residues are considered essential for the regulation and targeting of PM H^+^-ATPases. Phosphorylation of the C terminus of PM H^+^-ATPase provides binding sites for 14-3-3 proteins (Gaxiola et al., 2007; Falhof et al., 2016). Inactivated PM H^+^-ATPases usually exist as dimers and are converted into dodecamers when activated by phosphorylation of threonine on the C-terminal autoinhibitory domain and binding to 14-3-3 proteins (Olsson et al., 1998; Bobik et al., 2010; Falhof et al., 2016; Fuglsang and Palmgren, 2021). More interestingly, the quaternary structure of the yeast *Kluyveromyces lactis* PM H^+^-ATPase is a hexamer, and there is evidently a relationship between ATPase function and the aggregation state of the hexamer. The aggregated H^+^-ATPase hexamers are reportedly the activated state of the enzyme (Ruiz-Granados et al., 2019).

The protein kinases involved in PM H^+^-ATPase phosphorylation have not yet been identified (Falhof et al., 2016) and many details of PM H^+^-ATPase regulation are still unknown, although various studies have revealed some of the details of PM H^+^-ATPase regulation. It is well established that the fungal toxin fusicoccin is an effective activator of PM H^+^-ATPase (Marrè, 1979) and that vanadate is an inhibitor of PM H^+^-ATPase (Zhu et al., 2015). PKS5 blocks interaction between H^+^-ATPase and 14-3-3 proteins *via* phosphorylation of Ser-931in *A. thaliana* (Fuglsang et al., 2007). Small auxin UP-RNA proteins reportedly inhibit the activity of a family of type 2C protein phosphatases, which in turn modulates the Thr-947 phosphorylation status of PM H^+^-ATPases (Spartz et al., 2014). There is evidence of differences in regulation between different subfamilies. Bobik et al. (2010) reported that cold stress led to different PMA2 and PMA4 responses in *N. tabacum*, specifically that PMA2 was dephosphorylated within a few minutes, whereas PMA4 was not. Notably however, the regulatory mechanism distinguishing these two isoforms is unknown.

### Physiological Roles of PM H^+^-ATPase

PM H^+^-ATPase is active throughout the entire life process in plants, including seed germination, plant growth and defense, and pollen tube elongation, among other processes. According to acid growth theory PM H^+^-ATPase promotes proton efflux, acidifying the apoplast and facilitating the uptake of solutes and water, thus driving plant cell expansion and eventually resulting in hypocotyl elongation and root elongation (Spartz et al., 2014). AHA1 is reportedly a slow-wave potential regulator that activates the jasmonate defense pathway in distal leaves in response to wounding (Kumari et al., 2019). Recent reports indicate that there is a pH gradient in pollen tubes and that three members of the AHA family (AHA6, AHA8, and AHA9) are redundantly involved in the formation of a proton gradient and cell-wall patterning in the pollen tubes and are essential for polar growth in the pistil (Chen et al., 2020b; Hoffmann et al., 2020).

## PM H^+^-ATpase is the Limiting Factor in Light-Induced Stomatal Opening

### Function of Stomata

Stomata are a special structure on the surface of terrestrial plants that consists of a pore surrounded by two guard cells. Stomata control gas exchange between the atmosphere and plants, which is required for CO_2_ uptake in photosynthesis and the loss of H_2_O in transpiration. Plants can regulate the size of stomatal pores in response to environment changes, which can further coordinate photosynthesis with transpiration and also affect other physiological activities. Plant photosynthesis and transpiration are a significant component of global water and carbon cycles in atmospheric modeling. Excessive emission of CO_2_ by humankind has made the global carbon cycle imbalanced, causing a serious climate problem-global warming. Humankind is also facing a severe food crisis whereby globally a large number of people do not have access to enough food. As a result, stomata have drawn more attention, and improving the ability of plants to fix CO_2_ by manipulating stomata constitutes a potential means of addressing global climate changes and food insufficiency.

### Light-Induced Stomatal Opening

Light can stimulate stomatal opening *via* at least two mechanistically different pathways, the red-light pathway and the blue light pathway (Zeiger, 1983; Shimazaki et al., 2007). Blue light induces stomatal opening *via* a photosynthesis-independent signal pathway. A study in *Arabidopsis* suggests that blue light-induced stomatal opening is mediated by at least three key components: the blue light receptor phototropins, H^+^-ATPase, and plasma membrane inward-rectifying K^+^ channels (K^+^_in_ channels; Wang et al., 2014b). The phototropins phot1 and phot2 can be activated by blue light, and transmit signals downstream that activate PM H^+^-ATPase (Kinoshita et al., 2001). Activated PM H^+^-ATPase binds the 14-3-3 protein, phosphorylating the penultimate threonine on its C terminus, and pumps H^+^ out of the plasma membrane leading its hyperpolarization (Assmann et al., 1985; Shimazaki et al., 1986; Kinoshita and Shimazaki, 1999, 2002). It then evokes K^+^_in_ channels, promoting the uptake of K^+^ into the guard cells (Schroeder et al., 1987; Lebaudy et al., 2008). Simultaneously, some anions are required to compensate for the positively charged K^+^ in guard cells, mainly Cl^−^, malate^2−^, and NO^3−^ (Shimazaki, et al., 2007). Cl^−^ is taken up into the vacuole *via* the major vacuole chloride channel AtALMT9, which takes precedence over the accumulation of other ions (De Angeli et al., 2013). Malate^2−^ is required in the process, some of which is synthesized from starch degradation in the chloroplast, and some of which is transported in guard cells through the ABC transporter AtABCB14 (Shimazaki, et al., 2007; Lee et al., 2008). Blue light also induces starch degradation, which is activated *via* the phot1/phot2-dependent signaling pathway and associated with PM H^+^-ATPase activity, as evidenced by the observation that the *Arabidopsis* blue light-signaling mutants *phot1phot2* and *blus1* exhibit altered starch degradation in guard cells (Horrer et al., 2016). Some of the starch is used to produce malate^2−^, and some is converted to sucrose or hexose sugars which act as osmolytes or are used for respiration. The accumulated osmolytes including K^+^ and sugars reduce water potential and drive water uptake into the guard cell, significantly increasing its volume. Because the inner side of the guard cell wall is thicker than the outer side, the turgor of guard cells widens the stomatal pore and causes stomatal opening (Shimazaki et al., 2007).

Stomatal opening in response to red light can be inhibited by the photosynthetic electron transport inhibitor DCMU, indicating that it is related to photosynthesis (Sharkey and Raschke, 1981; Doi and Shimazaki, 2008; Wang et al., 2011; Suetsugu et al., 2014). Red light-induced stomatal opening is an indirect response to intercellular CO_2_ concentration. A high concentration of CO_2_ activates anion channels and outward-rectifying K^+^ channels (Brearley et al., 1997; Roelfsema et al., 2002). Red light promotes photosynthesis and reduces the intercellular CO_2_ concentration, relieving the negative effect of a high CO_2_ concentration on stomata opening. Conversely, a low CO_2_ concentration as a signal directly induces the protein kinase high leaf temperature 1, inhibiting S-type anion channels *via* convergence of blue light and CO_2_ (CBC)1 and CBC2 (Hashimoto et al., 2006; Hiyama et al., 2017). This promotes the uptake of K^+^ and stomatal opening. Under red-light illumination mesophyll cells and guard cells produce starch *via* photosynthesis, some of which is degraded into sucrose. Guard cells also take up sucrose from apoplasts. Sucrose can increase osmolytes and is used for the formation of ATP and malate^2−^ (Daloso et al., 2016). Photosynthesis in guard cells and mesophyll cells can also promote the accumulation of ATP, which is required for activated H^+^-ATPase (Tominaga et al., 2001; Wang et al., 2014a). Red light can reportedly induce photosynthesis-dependent phosphorylation of PM H^+^-ATPase in guard cells, which further promotes the absorption of K^+^ and stomatal opening. Notably however, neither sucrose nor CO_2_ as photosynthetic products are responsible for phosphorylation of H^+^-ATPase. It is currently unclear what mediates the photosynthesis-dependent phosphorylation of H^+^-ATPase (Ando and Kinoshita, 2018).

Although the details of signaling pathways involving phototropins and PM H^+^-ATPase have not been entirely clarified, the following important steps pertaining to relevant signal transduction have recently been reported as:

1. Phototropins function as blue light receptors *via* the chromophore flavin. In the N terminus of phototropins, there are two so-called light, oxygen, and voltage domains which act as binding sites for flavin mononucleotide and absorb blue light (Christie et al., 1999). After receiving a light signal phototropins are activated *via* autophosphorylation, with subsequent binding of 14-3-3 protein in response to blue light (Kinoshita et al., 2003). Blue light-activated phototropins are phosphorylated and transmit the signal downstream *via* the blue light-signaling 1 (BLUS1) protein kinase (Takemiya et al., 2013). It has also been reported that phototropins may be involved in another BLUS1-independent signaling pathway which inhibits stomatal opening by mediating dephosphorylation of PM H^+^-ATPase (Hosotani et al., 2021).
2. BLUS1 is a Ser/Thr protein kinase expressed in the cytoplasm of guard cells. It can be phosphorylated by phototropins and indirectly activate PM H^+^-ATPase, which transduces blue light signal from phototropins to type 1 protein phosphatase (PP1). The loss of blue light-dependent stomatal opening has been observed in BLUS1 mutants *via* infrared thermography (Takemiya et al., 2013). There is a Ser/Thr kinase domain in the N-terminal of BLUS1 and a regulatory domain in the C-terminal which inhibit its kinase activity. Phototropins phosphorylate Ser-348 within the C-terminal domain of BLUS1, alleviating auto-inhibition of the C-terminal domain and transmitting the signal downstream (Takemiya et al., 2013; Hosotani et al., 2021).
3. Blue light-dependent H^+^-ATPase phosphorylation (BHP) is a Raf-like protein kinase that indirectly activates PM H^+^-ATPase and functions in the cytosol of guard cells. It was identified by screening kinase inhibitors that suppress blue light-dependent H^+^-ATPase phosphorylation and stomatal opening in guard cells. It has been suggested that BHP can interact with both BLUS1 and PP1 *in vitro*, but only interaction between BHP and BLUS1 has been observed *in vivo* – thus whether or not BLUS1 phosphorylates BHP is uncertain (Hayashi et al., 2017).
4. PP1 and its regulatory subunit PRSL1 transmit signals between phototropin and PM H^+^-ATPase. The function of PP1 has been demonstrated by the transformation of guard cells with PP1c isoforms and inhibitors, which specifically reduces PP1 activity and suppresses stomatal opening. Tutomycin, an inhibitor of PP1, affects the phosphorylation of PM H^+^-ATPase but does not affect phototropins (Takemiya et al., 2006).

## Promotion and Upgregulation H^+^-ATpase Strategy Enhances Photosynthesis and Plant Growth

### Principles

Stomatal conductance is always proportional to the degree of stomatal aperture and is one of the main limiting factors in C3 photosynthesis (Farquhar and Sharkey, 1982), thus improving stomatal conductance is predicted to enhance photosynthesis and crop yield when there are no other stresses affect plant performance. Because stomatal conductance depends on both stomatal density and property, manipulations mainly focus on these two things. Sugano et al. (2010) reported that *STOMAGEN*, which encodes a secretory peptide involved in stomatal differentiation, regulates stomatal density. In that study overexpression of *STOMAGEN* in *Arabidopsis* increased stomatal density and enhanced the rate of photosynthesis by 30% compared to wild-type (WT) plants. Notably however, *STOMAGEN* overexpression also increased net water loss, and the whole plant biomass did not change (Tanaka et al., 2013). Similar results were observed in a mutant of *slow anion channel 1*, which had defects in the stomatal closing pathway resulting in constantly open stomata (Kusumi et al., 2012). These two methods successfully enhanced stomatal conductance and promoted photosynthesis rates, but significantly increased water loss was not beneficial with respect to accumulating biomass. From this perspective, increasing the aperture of stomata under light conditions and maintaining normal closure under stress and dark conditions are likely to be more favorable for plant growth.

**Figure 2:**
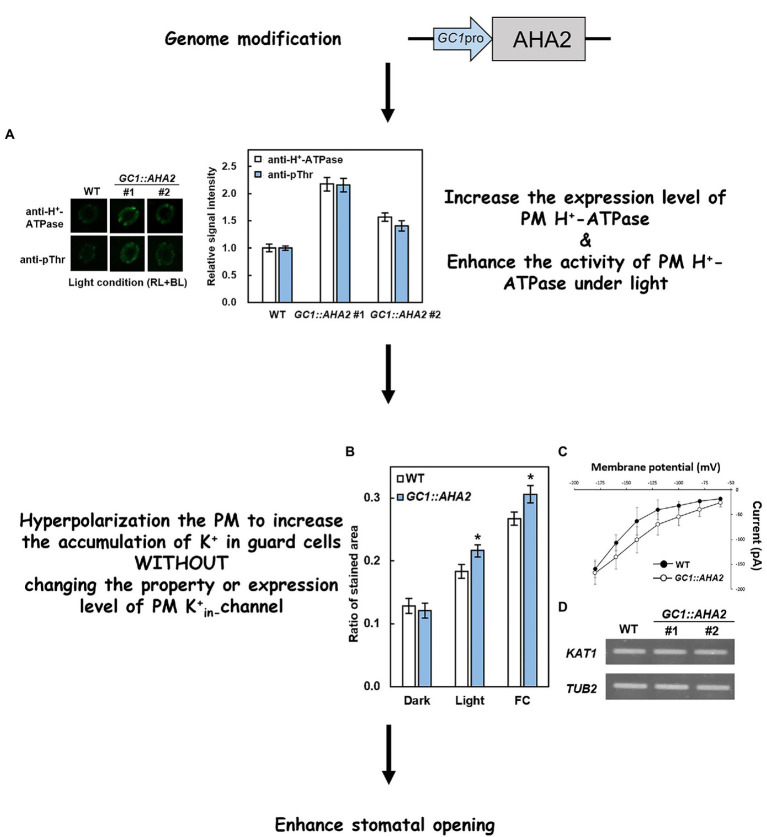
Principle of overexpression of PM H^+^-ATPase to enhance stomatal opening. **(A)** Immunohistochemical detection of PM H^+^-ATPase (anti-H^+^-ATPase) and its phosphorylation level (anti-pThr) in guard cells of WT and *GC1::AHA2* (the same lines used in Wang et al., 2014a). **(B)** K^+^ uptake in wild-type (WT) and *AHA2*-overexpressing guard cells. Bars indicate the average of three replicates, and error bars indicate the standard error (*n* = 3). Asterisks indicates a significant difference from WT (*p* < 0.05, Student’s *t*-test). **(C)** Current–voltage relationships in guard cell protoplasts of WT and *GC1::AHA2* detected by patch-clamp analysis. Solid circles represent WT, and hollow circles represent *AHA2*-overexpressing plants. Bars indicate the average of 4 replicates, and error bars indicate the standard deviation (*n* = 4 cells). **(D)** *KAT1* expression levels were comparable in WT plants and *AHA2*-overexpressing plants.

Recent studies have attempted to modify several key components involved in stomatal light responses to investigate their effects on stomatal traits and plant growth. As a component involved in stomatal opening induced by red and blue light, PM H^+^-ATPase reportedly has the potential to increase plant production *via* genetic modification. Wang et al. (2014a) used the strong guard cell promoter *GC1*, and Yang et al. (2008) overexpressed key genes in blue light-induced stomatal opening and reported that *AHA2* (*A. thaliana* H^+^-ATPase 2) was the limiting factor in this process. In *GC1::AHA2*-overexpressing plants, the levels of *AHA2* transcription and translation were increased, in conjunction with elevation of H^+^-ATPase phosphorylation (Figure 2A) and further hyperpolarization of the plasma membrane. Overexpression of PM H^+^-ATPase also affects the downstream K^+^ channel. *Via* sodium hexanitrocobaltate III staining of K^+^, it was determined that K^+^ uptake in *GC1::AHA2*-overexpressing plants was significantly higher than it was in WT plants under both light and exposure to fusicoccin conditions (Green et al., 1990; Figure 2B). The K^+^ channel current in *GC1::AHA2* was similar to that in WT at the same voltage however (Figure 2C), indicating that manipulating PM H^+^-ATPase in guard cells did not alter the properties of K^+^ channels. Moreover, the expression level of *potassium channel in Arabidopsis thaliana 1* (*KAT1*), a major guard cell K^+^ channel gene, also exhibited no significant change in *GC1::AHA2*-overexpressing plants (Figure 2D). Together these results indicated that the *GC1::AHA2*-overexpressing plant absorbed more K^+^ through the K^+^ channel without altering its expression level or properties. Eventually the ions and osmoticum accumulated, increasing the stomatal aperture.

Improving stomatal conductance is associated with increased photosynthetic rates and leads to greater biomass in *GC1::AHA2*-overexpressing plants. In light response curve studies, when light intensity was higher than 200 μmol m^−2^ s^−1^, stomatal conductance and the CO_2_ assimilation rate of *GC1::AHA2*-overexpressing plants were strikingly higher than those of WT. At a light intensity of nearly 200 μmol m^−2^ s^−1^, the growth of *GC1::AHA2*-overexpressing plants was increased, and the fresh and dry weights of rosettes and flower stems were significantly higher than those of WT (Wang et al., 2014a). The relationship between stomatal phenotypes and plant production has also been clarified. Because plants preferentially fix ^12^C during photosynthesis, an increase in stomatal conductance could lead to a decrease in the ^13^C/^12^C ratio (δ^13^C; Farquhar et al., 1989). *GC1::AHA2-*overexpressing plants had lower δ^13^C (Wang et al., 2014a), demonstrating that stomatal opening increased the intercellular CO_2_ concentration, which ultimately contributed to the plant growth. Moreover, it is reported that PM H^+^-ATPase also involved in plant root hair elongation and pollen tube growth (Axelsen and Palmgren, 2001; Hoffmann et al., 2019, 2020). Further studies are needed to exam whether *GC1::AHA2*-overexpression also affects root hair and pollen tube growth.

In one study, light-induced stomatal opening was disrupted by modifying certain components, but plant growth did not change. In *GC1::AHA2*-overexpressing plants with a Pro68-to-Ser point mutation at the AHA2 first transmembrane domain (*GC1::AHA2-P68S*), PM H^+^-ATPase was constitutively activated. *GC1::AHA2*-*P68S*-overexpressing plants exhibited continuous stomatal opening under light and dark conditions, and after the addition of abscisic acid (ABA), but there was no significant difference in plant growth compared to WT (Wang et al., 2014a). These results indicated that the functional integrity of PM H^+^-ATPase is essential for maintaining stomatal responses to light and promoting plant growth. Moreover, in plants overexpressing the stomatal opening positive regulator *flowering locus T*, stomata opened constantly under light and dark conditions, but closed normally with the application of ABA (Kinoshita et al., 2011). Compared with WT, *flowering locus T*-overexpression did not enhance plant growth (Wang et al., 2014a). These results may be partly due to the constant stomatal opening which led to lower water use efficiency, or it may be that PM H^+^-ATPase consumed ATP while the stomata remained open (Palmgren, 2001). Collectively, these results indicate that continuously open stomata under dark or stressed conditions probably hinder plant growth.

### Environmental Plasticity

The aforementioned transgenic plants were all cultivated under suitable environmental conditions. In sessile organisms however, the ability to adapt to environmental changes directly affects their probability of survival. Therefore, the responses of modified plants under various conditions are a useful indicator of their merits.

**Figure 3:**
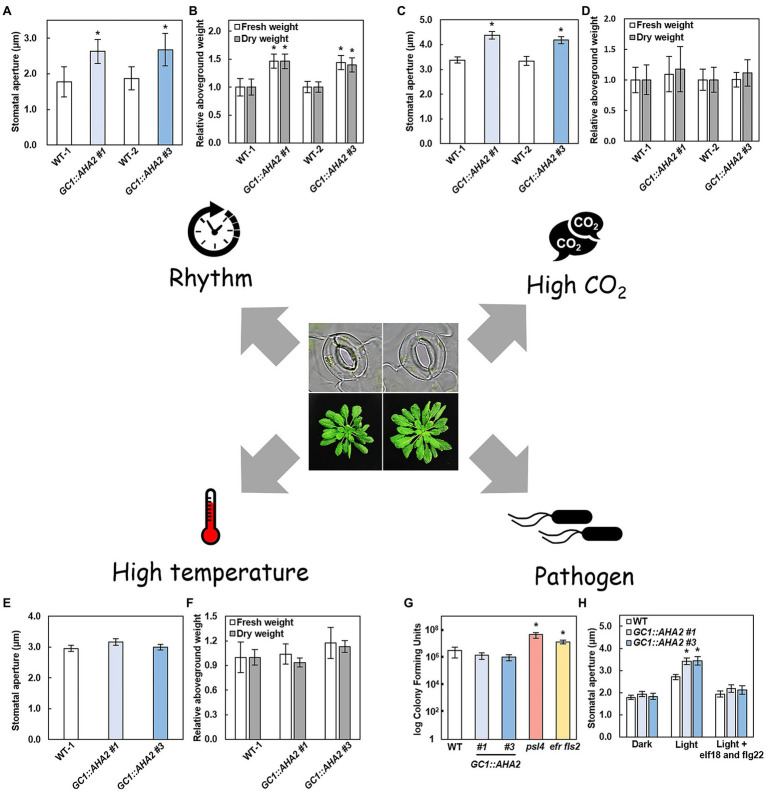
Environmental plasticity of *AHA2*-overexpressing plants. **(A–F)** Stomatal aperture **(A,C,E)** and relative aboveground weight **(B,D,F)** of WT and *AHA2*-overexpressing plants grown under short day (**A,B**, 8 h/16 h), high CO_2_ concentration (**C,D**, 800 ppm), and high temperature (**E,F**, 27°C) conditions. Bars indicate the average of 3 replicates, and error bars indicate the standard error (*n* = 3). Asterisks indicate a significant difference from WT (*p* < 0.05, Student’s *t*-test). **(G,H)** Responses of *AHA2*-overexpressing plants to biotic stress. **(G)** Colony-forming units of different plants. Four-week-old plants were sprayed with a *Pst* DC3000 suspension (OD600 of 1) in 10 mM MgCl_2_ with 0.02% silwet L-77. Leaf surfaces were sterilized with 70% ethanol for 1 min, then washed with water twice. Four leaf disks were ground in 1 ml of 10 mM MgCl_2_. The mutant plants *psl4* and *efr fls2* are insensitive to *Pst* DC3000. **(H)** Stomatal responses to bacterial epitopes. The concentration of elf18 and flg22 were 5 μM, respectively. Bars indicate the average of three independent samples with two replicates each, and error bars indicate the standard deviation (*n* = 3). Asterisks indicate a significant difference from WT (*p* < 0.01, Student’s *t*-test).

Plants always face different environmental conditions, such as changes in day length and light conditions over time, high CO_2_ conditions due to increased global CO_2_ concentrations. The performance of *GC1::AHA2*-overexpressing plants under these conditions warrants attention. Under short-day conditions, the stomatal aperture of *GC1::AHA2*-overexpressing plants was notably higher than that of WT, as was their fresh and dry aboveground weight (Figures 3A,B). The stomatal aperture of the long-day plant *A. thaliana* was significantly reduced when the daylight duration was shortened (Aoki et al., 2019). Therefore, the enlarged stomatal aperture in *GC1::AHA2*-overexpressing plants may compensate for its reduction. The light intensity in this experiment was suitable (150 μmol m^−2^ s^−1^), which led to the stomatal aperture becoming the main limiting factor of photosynthesis under light. Therefore, the increased CO_2_ absorption through open stomata ultimately increased the biomass of the plants. Under low light conditions, the production of transgenic plants was identical to that of WT, although the stomatal aperture increased (Wang et al., 2014a). It may be that intercellular CO_2_ concentration affected the photosynthetic rate under low light, but electron transport was still a limiting factor in this process (Farquhar et al., 1980). Similarly, under elevated CO_2_ conditions, *GC1::AHA2*-overexpressing plants also exhibited increased stomatal apertures and an unchanged biomass (Figures 3C,D). It may be that stomatal restriction was not the main limiter of intracellular CO_2_ concentration (Farquhar et al., 1980; Busch et al., 2020).

Because *AHA2* overexpression alters stomatal traits, the biotic and abiotic stress associated with stomata may affect plant traits. The most deleterious abiotic sources of stress in plants are drought and heat. Under severe drought stress, plants mainly produced the phytohormone ABA to induce stomatal closure (Roelfsema et al., 2004). As well as inducing the activation of R-type and S-type anion channels, ABA also inhibited the capacity of PM H^+^-ATPase to maintain membrane depolarization which led to stomatal closure (Goh et al., 1996; Roelfsema et al., 2004). *GC1::AHA2*-overexpressing plants exhibited more PM H^+^-ATPase expression but were still regulated by ABA, resulting in the same stomatal and biomass phenotypes as WT. Under mild drought conditions, *GC1::AHA2*-overexpressing plants also exhibited the same performance as WT (Wang et al., 2014a). When land plants are exposed to high temperature they generally facilitate leaf cooling by opening stomata to increasing transpiration. It has been reported that components of the blue light-signaling pathway, including PM H^+^-ATPase and its upstream and downstream substances, are involved in high-temperature-induced stomatal opening (Kostaki et al., 2020). Notably however, the phenotype of *GC1::AHA2*-overexpressing plants was the same as WT (Figures 3E,F). It may be that the stomatal aperture of WT also increased at high temperature, eliminating the difference between them (Rogers, 1979).

The main source of biotic stress in plants is pathogen infection. One form of plant defense against bacterial invasion is closing stomata (Melotto et al., 2006). When *GC1::AHA2*-overexpressing plants were sprayed with *Pst* DC3000 (*Pseudomonas syringae* pv. *tomato* DC3000), the number of pathogenic bacteria entering the leaves through stomata remained unchanged compared to WT, indicating that the *GC1::AHA2*-overexpressing plants were normally susceptible to pathogens (Figures 3G,H). After adding a mixture of the bacterial epitopes elf18 and flg22, the stomatal apertures of the *GC1::AHA2*-overexpressing plants were similar to those of WT. This may be a result of a normal phytohormone pathway (such as the ABA pathway) in *GC1::AHA2*-overexpressing plants, which regulated stomatal closure to inhibit the entry of pathogenic bacteria. These results indicate that transgenic plants have a normal capacity to resist biotic stress.

Collectively, the above-describe observations suggest that under favorable conditions, especially when the stomatal aperture is the main limiting factor of photosynthesis, the CO_2_ assimilation rate and plant production of *GC1::AHA2*-overexpressing plants may be superior to WT. Under stress conditions, whether it was biotic or abiotic, *GC1::AHA2*-overexpressing plants showed neither advantages nor inferiority, and grew as well as WT (Table 1). These advantages of the technology enable it to be used in combination with other methods to further enhance photosynthesis and plant growth.

**Table 1:** Summary of PM H^+^-ATPase-overexpression plant characteristics under different conditions.

Condition	Stomatal aperture	Plant growth
Well condition^*^ (High light, long day, well watered, ambient CO_2_)	Increase	Increase
Short-day	Increase	Increase
Low light^*^	Increase	Normal as WT
High CO_2_	Increase	Normal as WT
Drought^*^	Normal as WT	Normal as WT
High temperature	Normal as WT	Normal as WT
Pathogen	Normal as WT	-

WT means the wide type of *Arabidopsis thaliana*.

* Data are from Wang et al. (2014a).

## Applications of Promotion and Upgregulation H^+^-ATpase Plants

### Overexpression of PM H^+^-ATPase Promotes Grain Yield in Rice

*O. sativa* is one of the most important crops worldwide and is the staple food of 3 billion people (Woolston, 2014). As a monocotyledon its stomata are formed as pairs of dumbbell-shaped guard cells surrounded by two subsidiary cells. Because of the difference in guard cell morphology between rice and *Arabidopsis*, whether modifying PM H^+^-ATPase could lead to a breakthrough in crop production warrants investigation.

There are 10 isoforms of OSA located on the plasma membrane, most of which are involved in light-induced stomatal opening. Like PM H^+^-ATPase in kidney-shaped guard cells, the activity of most OSAs is regulated *via* phosphorylation of penultimate threonine. Among them, OSA7 is the dominant isoform in rice guard cells and plays an indispensable role in plant growth. The stomatal conductance of the mutant *osa7* was significantly lower than that of WT, and plant growth was severely restricted – to the extent that the plants were unable to develop to the reproductive stage. OSA7 is involved in blue light-induced stomatal opening (Toda et al., 2016). Other homologous genes involved in the blue light-signaling pathway in kidney-shaped guard cells have also been identified in rice (Kanegae et al., 2000; Takemiya et al., 2013), but the specific components and mechanism of the pathway in dumbbell-shaped guard cells remain to be determined.

OSA1 is one of the main isoforms in rice. A recent study on *osa1* mutants and overexpressing rice plants improved our understanding of *OSA* genetically modified plants (Zhang et al., 2021). Because there is no guard cell-specific promoter in rice Zhang et al. (2021) used the *CaMV-35S* promoter to constitutively express *OSA1*. Stomatal conductance and the CO_2_ assimilation rate of *OSA1*-overexpressing plants were significantly higher than those of WT. Because active PM H^+^-ATPase could facilitate the absorption of ammonium (NH_4_^+^) in root, the NH_4_^+^ absorption rate in *OSA1*-overexpressing plants was also significantly increased. Moreover, because NH_4_^+^ absorption and assimilation occur almost simultaneously in plant roots (Xu et al., 2012), compared with both WT and mutants, the total amount of nitrogen in *OSA1*-overexpressing plants also increased (Zhang et al., 2021). Carbon and nitrogen are both associated with the rate of photosynthesis in leaves and root nitrogen assimilation, and both were significantly increased in *OSA1*-overexpressing plants.

The above-mentioned physiological changes were only short-term processes, and the final change in plant biomass also depended on a series of downstream genes. Long-term cultivation of *OSA1*-overexpressing plants increased the expression of genes associated with plant growth. Remarkably, *via* differentially expressed gene analysis, it was determined that overexpressing PM H^+^-ATPase activated a series gene involved in ammonia assimilation, amino acid metabolism, photosynthesis, and GRF4, a key transcription factor that integrates nitrogen assimilation, carbon fixation, and plant growth in rice (Li et al., 2018; Zhang et al., 2021). Positive feedback between the physiological process and gene expression eventually results in the crop yield of *OSA1*-overexpressing plants under different fertilization conditions being notably higher than that of WT, indicating that overexpression of PM H^+^-ATPase in rice has the potential to facilitate reduced use of nitrogen fertilizer. It also provides a new direction for the coordination of the N and C assimilation pathway, and to emphasize the importance of PM H^+^-ATPase (also known as proton pump), Zhang et al. (2021) suggested to define the “promotion and upregulation of PM H^+^-ATPase plants” as “PUMP” plants.

### Nanomaterials Benefit Plants by Increasing PM H^+^-ATPase Expression

Nanomaterial science can be used to increase the expression of PM H^+^-ATPase in plants, and its effects are similar to those of genetic manipulation. Engineered nanomaterials have been widely applied in environmental remediation, agriculture, and other fields, and have specific effects on PM H^+^-ATPase in plants. The effects of engineered nano zerovalent iron (nZVI) on PM H^+^-ATPase have been investigated. In studies on nZVI in *Arabidopsis*, the surface of nZVI was oxidized to iron oxide-hydroxide, which was hydrolyzed resulting in OH^−^ and increased rhizosphere pH, and the activation and expression of PM H^+^-ATPase were promoted. Remarkably, the treated plants maintained satisfactory water use efficiency while increasing the stomatal aperture (Kim et al., 2015). In another study, nZVI was investigated in cucumber plants, and under favorable conditions, the photosynthetic capacity of plants was enhanced (Yoon et al., 2019). Moreover, when carbon nanoparticles were added to tobacco plant cell suspensions the expression of PM H^+^-ATPase was significantly upregulated, and plant growth was also increased (Chen et al., 2020a). These technologies constitute a potential new method for increasing PM H^+^-ATPase in plants to improve plant production.

## Conclusion

PM H^+^-ATPase occurs in numerous plants, especially crops, and PM H^+^-ATPase manipulation has a wide range of potential applications in various crops under certain conditions. In the future, the application of gene-editing tools, such as the Crispr/cas9 system to edit the promoter region of PM H^+^-ATPase to enhance PM H^+^-ATPase expression, may facilitate the breeding of non-transgenic promotion and upregulation H^+^-ATPase plants. Chemical compounds that induce PM H^+^-ATPase activity, such as plant hormones, auxin (Takahashi et al., 2012), and brassinosteroid (Minami et al., 2019), and stomatal opening (Toh et al., 2018) is also candidates for incorporation into promotion and upregulation H^+^-ATPase strategies.

## Author Contributions

YW and TK conceived the idea. YW, TF, and ZR conducted experiments and performed data analyses. ZR, BS, DY, and YW wrote the manuscript. YS, TK, and YW reviewed and edited the manuscript. All authors contributed to the article and approved the submitted version.

## Funding

This study was financially supported by the National Natural Science Foundation of China (grant number 31972937 to YW), the Youth Fund of the Ministry of Education Laboratory for Earth Surface Processes, Peking University (to YW), Grants-in-Aid for Scientific Research from MEXT (20H05687 and 20H05910 to TK), and the Advanced Low Carbon Technology Research and Development Program from the Japan Science and Technology Agency (JPMJAL1011 to TK).

## Conflict of Interest

The authors declare that the research was conducted in the absence of any commercial or financial relationships that could be construed as a potential conflict of interest.

## Publisher’s Note

All claims expressed in this article are solely those of the authors and do not necessarily represent those of their affiliated organizations, or those of the publisher, the editors and the reviewers. Any product that may be evaluated in this article, or claim that may be made by its manufacturer, is not guaranteed or endorsed by the publisher.
